# A41 FONTAN ASSOCIATED LIVER DISEASE: THE ROLE OF TRANSIENT ELASTOGRAPHY IN CHILDREN AND ADOLESCENTS

**DOI:** 10.1093/jcag/gwac036.041

**Published:** 2023-03-07

**Authors:** L Khendek, V Molina, N Laverdure, K Abukasm, S Khullar, M Lachaud, J Dubois, D Dal Soglio, J Miro, A Fournier, M Paganelli

**Affiliations:** 1 Gastroenterology, Hepatology and Nutrition, Sainte-Justine University Hospital Center; 2 University of Montreal; 3 Cardiology; 4 Pathology; 5 Radiology, Sainte-Justine University Hospital Center, Montreal, Canada

## Abstract

**Background:**

Non-invasive assessment of Fontan Associated Liver Disease (FALD) is of interest, but studies have yielded inconsistent results about the correlation of severity of disease with laboratory values and imaging. Transient elastography (TE) is a non-invasive imaging modality used commonly for liver stiffness measurement (LSM) and in Fontan patients it is hypothesized to reflect not only liver fibrosis but also venous congestion.

**Purpose:**

To better define the potential role of TE for non-invasive assessment of the severity of FALD.

**Method:**

This was a retrospective study conducted on patients’ medical records at CHU Sainte-Justine Hospital. Patients less than 18 years of age with FALD who had at undergone at least one LSM by TE between 1998-2021 were included. The relationship between LSM and liver function tests, hepatic ultrasound findings (including a cirrhosis score), cardiac catheterization results and histological fibrosis scores were analyzed. The impact of interventions during cardiac catherization on LSM were also studied.

**Result(s):**

A total of 54 patients (36 boys and 18 girls) with FALD were studied. Median age at Fontan surgery was 4.6 years (IQR 4.0 ─ 5.4 years). Higher LSM values significantly correlated with longer time from Fontan, higher total and direct bilirubin and GGT levels, higher INR, longer APTT, lower Factor V, and lower absolute lymphocyte count. Greater LSM was also significantly associated with the presence of heterogenous parenchymal echogenicity, irregular liver contours and greater ultrasonographic cirrhosis scores. Higher TE values were significantly correlated with higher wedged hepatic venous pressure and Fontan pressure. After catherization interventions that addressed stenoses, there was a statistically significant reduction in mean LSM (24.9±3.63 kPa vs 15.8±4.6 kPa, *p*=0.005). After closure of significant pulmonary collaterals, mean LSM tended to increase, but this difference did not reach statistical significance (19.1±1.9 kPa vs 24.6±3.5 kPa, *p*=0.2). At liver biopsy, significant direct correlation was found between LSM and the grade of sinusoidal fibrosis and LSM. TE with values >20 kPa were found to have higher grades of sinusoidal fibrosis, while values <20kPa had higher grades of sinusoidal dilatation.

**Conclusion(s):**

This study showed that TE allows to identify patients with higher cholestatic parameters, more severe liver fibrosis at biopsy and sonographic signs suggestive of cirrhosis. Moreover, it confirmed that liver congestion significantly contributes to LSM values. Interestingly, catheter interventions addressing pulmonary stenoses led to the improvement of TE measurements, giving hope for the reduction of hepatic venous congestion in these patients, which might have an effect on their FALD. Finally, the LSM threshold of 20 kPa could be useful clinically as a value above which fibrosis is likely to be significant, while if below could indicate a greater contribution from hepatic congestion.

**Please acknowledge all funding agencies by checking the applicable boxes below:**

None

**Disclosure of Interest:**

None Declared

